# A Study on the Depressive Symptoms and Life Satisfaction Among Middle-Aged and Older Adults in China Based on CHARLS 2020 Data

**DOI:** 10.3390/bs16081455

**Published:** 2026-08-21

**Authors:** Shu Wang, Yoshihisa Shirayama, Ishtiaq Ahmad, Miyoko Okamoto, Motoyuki Yuasa

**Affiliations:** Department of Global Health Research, Graduate School of Medicine, Juntendo University, Tokyo 113-8421, Japan

**Keywords:** depressive symptoms, mental health, Chinese middle-aged and older adults, lifestyle, healthy behaviors

## Abstract

Background: Depressive symptoms have become a significant public health issue faced by middle-aged and older adults in China. Although previous studies have primarily focused on demographic and socioeconomic factors, the association between modifiable lifestyle behaviors and depressive symptoms still requires further investigation. Objective: To examine the associations between sleep duration, physical activity, digital participation, social participation, and depressive symptoms as well as life satisfaction among middle-aged and older adults in China. Methods: A cross-sectional analysis was conducted using data from the 2020 China Health and Retirement Longitudinal Survey (CHARLS). Depressive symptoms were defined as CES-D-10 scores ≥10, with 13,576 individuals included in the analysis sample. Adjusted prevalence ratios (aPRs) were estimated using modified Poisson regression, while ordinal logistic regression was employed to analyze life satisfaction. The analysis controlled for covariates such as demographics, socioeconomic status, and health status, and accounted for the complex sampling design. Results: The weighted prevalence of depressive symptoms was 44.5%. Sleep deprivation (<7 h/day) was significantly associated with elevated depressive symptoms (aPR = 1.49, 95% CI: 1.33–1.68); participation in recreational activities was significantly associated with reduced depressive symptoms (aPR = 0.86, 95% CI: 0.77–0.96); adequate physical activity was associated with a higher prevalence of elevated depressive symptoms (aPR = 1.17, 95% CI: 1.04–1.32); watching videos (aPR = 0.87, 95% CI: 0.76–0.99) and playing online games (aPR = 0.65, 95% CI: 0.50–0.83) were significantly associated with reduced depressive symptoms; and overall internet use was correlated with lower life satisfaction (OR = 0.90, 95% CI: 0.81–1.00). Women, rural residents, and low-income groups exhibited higher prevalence of elevated depressive symptoms. Conclusion: Sleep deprivation, as well as different types of social engagement, physical activity, and digital behavior, exhibit distinct associations with depressive symptoms in middle-aged and older adults. Behavioral intervention strategies targeting sleep hygiene, leisure activities, and tailored digital behaviors warrant further attention.

## 1. Introduction

Global population aging is accelerating, and China is undergoing one of the most rapid demographic transformations worldwide (Kemoun et al., 2022). As the country with the largest population of older adults, China’s population aged 60 years and older exceeded 260 million in 2020, accounting for 18.7% of the total population. With the birth cohort from the “Second Baby Boom” period (1962–1975) gradually reaching retirement age starting in 2022, China’s population aging process is expected to accelerate further, imposing unprecedented pressure on family care systems and social pension security frameworks (X. Chen et al., 2022). Forecasts indicate that China’s total population will decline from 1.4 billion in 2017 to 732 million by 2100 (95% confidence interval: 456–1.499 billion), representing a reduction of 48.0%; by 2050, China’s total fertility rate is likely to remain below the replacement level of 2.1, coexisting with accelerated aging and fundamentally reshaping the nation’s demographic structure and socio-economic development landscape (Vollset et al., 2020).

Population ageing creates persistent psychological stress for middle-aged and older adults, which was worsened by COVID-19 containment policies (Oh et al., 2024). While internet access eased pandemic distress particularly for rural older adults, its protective influence fluctuated throughout the pandemic (X. Wang & Ye, 2024; Wu et al., 2025). Travel restrictions greatly limited opportunities for offline socialising and leisure activities, leading to marked declines in middle-aged and older adults’ satisfaction with interpersonal contact (Schlomann et al., 2021). Life satisfaction serves as a core indicator for evaluating psychological status during the pandemic. Relevant statistics show that individuals with low life satisfaction have a 42.9% higher prevalence of mental comorbidities (Y. L. Chen et al., 2025). Although prolonged isolation exerted adverse impacts on health behaviours such as physical activity, it also fostered more informal mutual support within families (Ates Bulut et al., 2024). Despite evidence confirming leisure activities act as a key mediator between social media use (e.g., WeChat) and depressive symptoms (G. Wang et al., 2023), few national cohort studies jointly examine sleep, activity, social and digital behaviours against depression and life satisfaction.

Against the backdrop of this demographic transition, depressive symptoms are relatively common among older adults in China, yet they often remain underrecognized and inadequately addressed (Zhou et al., 2023). Geriatric depression is closely associated with declining quality of life, functional decline, increased mortality risk, and higher healthcare expenditures (Gambaro et al., 2022; P. Zhang et al., 2023). Its impacts involve both biological and sociological pathways: biologically, geriatric depression is frequently accompanied by neuroinflammation, impaired immune function, and coexistence of multiple chronic diseases, accelerating functional decline; sociologically, depression can lead to social isolation, reduced healthcare-seeking behavior, and decreased treatment adherence, further exacerbating health outcomes and burdening the healthcare system.

Although the impact of demographic and clinical factors on depression has been extensively studied, the role of modifiable lifestyle and behavioral factors in middle-aged and older adults remains underexplored. Recent evidence suggests that regular physical exercise, adequate sleep, and active social engagement may be associated with better mental health outcomes and promote overall health and well-being (Kim et al., 2022; Plante, 2021; Zhu et al., 2024). Concurrently, “digital participation”—defined as using the internet for communication, entertainment, or managing daily tasks—has become increasingly prevalent among middle-aged and older adults and may yield additional psychological and social benefits (Alber et al., 2023). However, few studies have systematically evaluated the comprehensive impact of these behavioral factors on mental health and subjective well-being based on nationally representative data; existing research is often limited by small sample sizes, inconsistent definitions, or a focus on younger populations (Eichstaedt et al., 2020).

To address the aforementioned shortcomings, this study utilized data from the 2020 China Health and Retirement Longitudinal Study (CHARLS) to analyze the associations between sleep duration, physical activity, digital participation, social engagement, and depressive symptoms and life satisfaction among middle-aged and older adults, aiming to provide empirical evidence for developing behavior-oriented mental health promotion strategies in the context of an aging society.

## 2. Materials and Methods

### 2.1. Data Sources and Study Population

**Figure 1:**
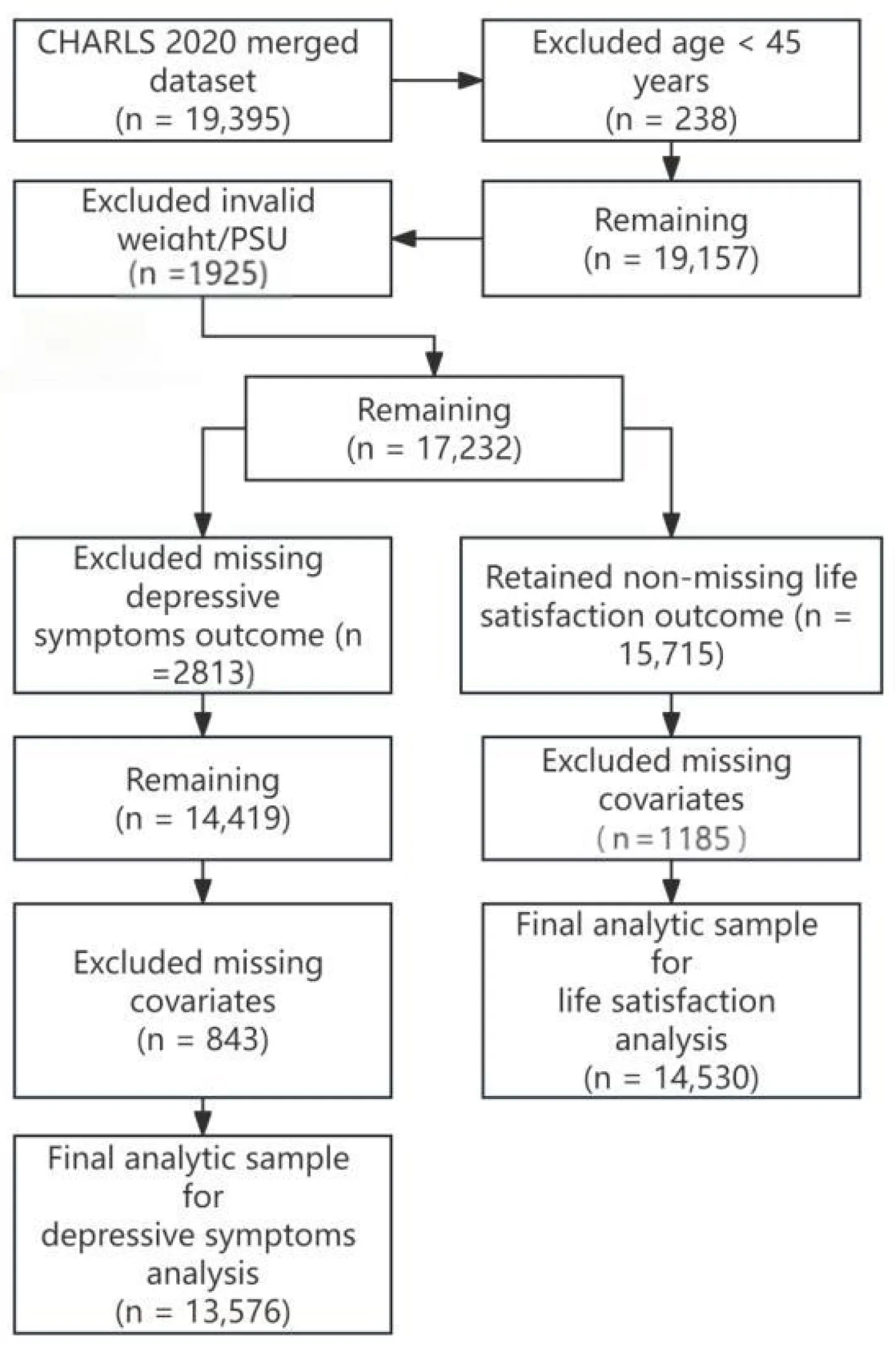
Participant flow diagram for the CHARLS 2020 analysis.

This study utilized the 2020 survey data from the China Health and Retirement Longitudinal Survey (CHARLS). CHARLS is a nationally representative longitudinal survey conducted among Chinese adults aged 45 and older, employing a multi-stage stratified probability sampling method that covers 28 provinces, autonomous regions, and municipalities directly under the central government. The sample screening process is illustrated in Figure 1 and Table 1: the merged CHARLS 2020 dataset comprised 19,395 individuals; 238 individuals aged <45 years were excluded; 1925 individuals lacking valid sampling weights or primary sampling unit (PSU) information were excluded; 2813 individuals with missing depression symptom outcomes were excluded; and finally, 843 individuals with missing covariates were excluded, resulting in a valid sample size of 13,576 individuals for the depression symptom analysis. In this study, responses that significantly deviated from reasonable ranges—such as sleep duration <1 h or >24 h, or physical activity MET values > 20,000—were defined as outliers and excluded. The sample for the life satisfaction analysis consisted of 14,530 individuals. The original CHARLS study was approved by the Ethics Committee of Peking University (IRB number: IRB00001052-14015), and all respondents provided written informed consent. This study constitutes a secondary analysis of publicly available database data and does not require additional ethical approval.

### 2.2. Measures

#### 2.2.1. Primary Outcome Measure

The primary outcome was depressive symptoms, assessed using the 10-item simplified version of the Center for Epidemiological Studies Depression Scale (CES-D10) (Pan et al., 2022). Respondents were asked about the frequency of occurrence of each of the 10 depressive symptoms over the past week, with each item scored on a four-point scale ranging from 0 to 3, yielding a total score ranging from 0 to 30. In this study, a CES-D10 score ≥10 was defined as clinically significant elevation of depressive symptoms (Pan et al., 2022). This cutoff value has been well validated among Chinese adults aged 45 years and older (Fu et al., 2022). In sensitivity analyses, the CES-D10 score was also included as a continuous variable in the linear regression model.

#### 2.2.2. Secondary Outcome Measure

Life satisfaction was retained as the secondary outcome because it directly reflects subjective well-being and was conceptually aligned with the study objective. Self-rated health was removed because it represents a broader assessment of general health rather than psychological well-being and substantially overlaps with the health-status covariates included in the models. The secondary outcome measure was life satisfaction, assessed using the “Do you feel satisfied with your life?” item from the CHARLS questionnaire, which employs a five-point Likert scale: 0 = completely dissatisfied, 1 = somewhat dissatisfied, 2 = relatively satisfied, 3 = very satisfied, and 4 = extremely satisfied (Y. Zhao et al., 2023). This variable was retained as an ordered five-category outcome and analyzed using ordinal logistic regression to fully leverage the ordinal information between the different levels. A parallel lines test was conducted to verify the applicability of the proportional odds assumption.

#### 2.2.3. Key Independent Variables

Sleep duration was defined as self-reported total daily sleep minutes and categorized into three groups: <7 h/day, 7–9 h/day (reference group), and >9 h/day (Song et al., 2024). Physical activity was assessed using the Short International Physical Activity Questionnaire (IPAQ), with the weekly total physical activity level calculated in MET-minutes/week; based on the World Health Organization (WHO) recommendations, participants were classified into two categories: insufficient (<600 MET-minutes/week, reference group) and adequate (≥600 MET-minutes/week) (Shan et al., 2025). It should be noted that the CHARLS physical activity measurement encompassed three dimensions: work-related activities (including agricultural work), transportation, and leisure exercise. Smoking status was categorized as current smoker or non-smoker. Alcohol consumption status was categorized as no alcohol consumption (reference group), consuming alcohol less than once per month, or consuming alcohol at least once per month (Peng et al., 2024). Social participation was defined as participation in any of the following five types of activities during the survey period, based on CHARLS questionnaire items (binary: yes/no, without requiring a minimum frequency): (1) interacting with friends or relatives; (2) leisure and recreational activities; (3) community-based organizational activities; (4) volunteer or charitable activities; and (5) participation in training or educational activities. Digital participation was assessed based on internet usage patterns and specific online activities (such as chatting, reading news, watching videos, or playing online games), each treated as a binary variable; individuals who did not use these specific functions (including non-internet users) served as the reference group.

#### 2.2.4. Covariates

**Figure 2:**
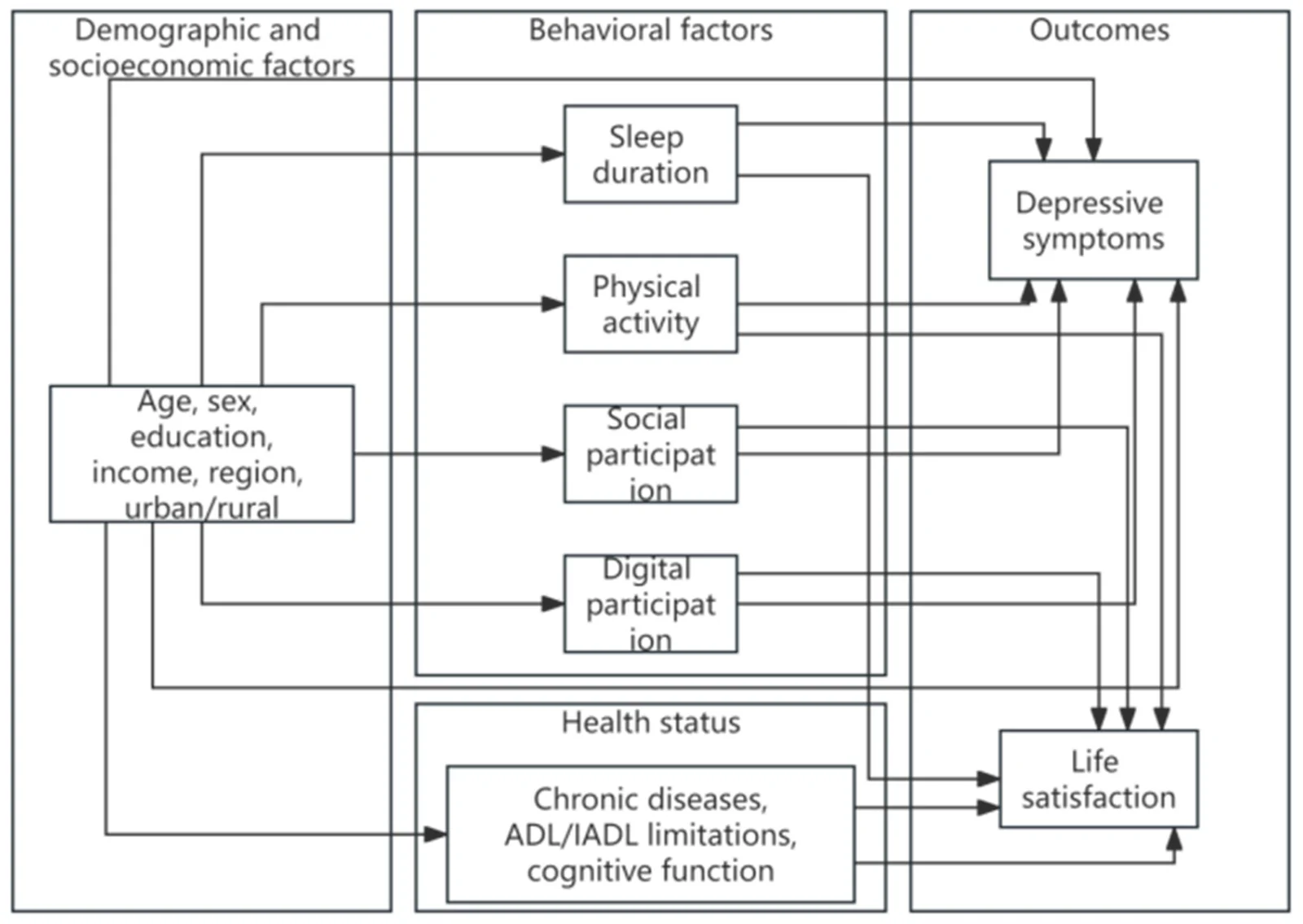
DAG-informed conceptual framework for covariate selection.

The selection of covariates was based on a directed acyclic graph (DAG) framework; see Figure 2 and Table 2: (1) Demographic and socioeconomic covariates included age group (45–59 years, 60–74 years, ≥75 years), sex, marital status (married/partnered vs. unmarried, including widowed, divorced, and separated), education level (primary school or below/secondary school/college degree or above), annual individual income, region (Northeast China/East China/Central China/West China), urban or rural residence, type of health insurance, number of household members, and number of living children. Annual individual income was calculated by summing income from employment, pensions, government transfers, and other individual sources. It was categorized into quintiles based on the survey-weighted income distribution among design-eligible participants, with Q1 representing the lowest-income group and Q5 the highest-income group. (2) Health status: number of chronic diseases, number of limitations in activities of daily living (ADLs), and number of limitations in instrumental activities of daily living (IADLs). (3) Cognitive function: core cognitive score. All aforementioned health and cognitive variables were treated as baseline characteristics in this cross-sectional study and incorporated into the model.

### 2.3. Statistical Analysis

All statistical analyses were performed using Stata/MP 19 software (StataCorp, 2025), strictly employing the individual sampling weights, stratification variables, and PSU information provided by CHARLS, with adjustments for complex sampling designs using the svy: command. A two-sided *p*-value <0.05 was considered statistically significant. In descriptive analysis, continuous variables were expressed as weighted mean ± standard deviation, while categorical variables were presented as weighted percentages. The primary analysis of depressive symptoms employed modified Poisson regression with robust standard errors to estimate adjusted prevalence ratios (aPRs) and their 95% confidence intervals (CIs). Given that depressive symptoms accounted for approximately 45% of cases in this study—a common outcome—the modified Poisson regression directly estimated prevalence ratios, avoiding overestimation of association strength through odds ratios (ORs). All exposure variables and covariates were included in the models, with variance inflation factors (VIFs) reported to assess multicollinearity. The primary analysis of life satisfaction utilized ordinal logistic regression to estimate cumulative odds ratios (ORs) and 95% CIs, with parallel lines test applied to evaluate the proportional odds assumption. Subgroup analyses were conducted by sex and age group, with interaction terms evaluated using the Wald test. Sensitivity analyses included: (1) comparisons between baseline-adjusted and fully adjusted models; (2) separate modeling of each item of digital participation; (3) inclusion of CES-D10 scores as a continuous variable in linear regression; and (4) use of multiple imputation for missing data.

## 3. Results

### 3.1. Participant Characteristics

The depression symptom analysis included 13,576 respondents, with the weighted sample representing the Chinese middle-aged and older adults aged 45 and above. After weighting, the mean age was 61.3 years (standard deviation = 9.5), with women accounting for 50.2% and men for 49.8%. The age distribution was as follows: 45–59 years old accounted for 46.6%, 60–74 years old accounted for 42.8%, and those aged 75 and above accounted for 10.6%. In terms of socioeconomic characteristics, rural residents accounted for 46.8%, individuals with an education level of primary school or below accounted for 55.7%, and those in the lowest quintile of income accounted for 24.9%. After weighting, the prevalence rate of depressive symptoms (CES-D10 ≥ 10) was 44.5%, with the mean CES-D10 score being 5.98 (standard deviation = 5.67). Complete descriptive statistics are presented in Table 3.

The life satisfaction analysis sample consisted of 14,530 individuals with characteristics consistent with the main sample. The weighted average age was 61.7 years (standard deviation = 9.7); females accounted for 51.1% and males for 48.9%. The age distribution was as follows: 45–59 years (45.0%), 60–74 years (43.2%), and 75 years or older (11.8%). Rural residents constituted 47.6%, while those with an education level of primary school or below represented 57.7%. The mean life satisfaction score was 2.27 (out of 4; standard deviation = 0.77). Detailed results are presented in Table 4.

To assess potential selection bias, we compared the included participants with design-eligible participants excluded due to missing data (*n* = 3656). The excluded group was older, more often female, and had slightly higher chronic disease counts and CES-D10 scores (all *p* < 0.001); however, effect sizes were modest. Detailed comparisons are shown in Supplementary Table S4.

### 3.2. Univariate Analysis

Table 5 presents univariate associations between each factor and depressive symptoms as well as life satisfaction. The survey-adjusted Wald F tests revealed that depressive symptoms were significantly associated with sex, income quintiles, region, marital status, type of health insurance, education level, urban or rural residence, sleep duration, adequate physical activity, smoking, alcohol consumption, social interactions with friends and relatives, leisure and recreational activities, community activities, volunteer/civic activities, participation in training or educational activities, and internet use (all *p*-values for the F-test were <0.05). Specifically, the prevalence of elevated depressive symptoms was 53.8% among females versus 35.0% among males; it was 49.3% among rural residents versus 40.2% among urban residents; and when categorized by sleep duration, the rates were 54.9% in the <7 h group, 39.0% in the 7–9 h group, and 44.2% in the >9 h group. Regarding life satisfaction, the univariate analysis showed significant associations between life satisfaction and age group, income quintiles, type of health insurance, education level, sleep duration, alcohol consumption, and internet use (*p* < 0.05). The specific F-values and *p*-values are detailed in Table 5.

### 3.3. Multifactorial Adjusted Poisson Regression for Depressive Symptoms

Table 6 presents the adjusted prevalence ratios (aPRs) from modified Poisson regression. In the fully adjusted model, sleep duration <7 h/day was significantly associated with elevated depressive symptoms (aPR = 1.49, 95% CI: 1.33–1.68, *p* < 0.001), whereas sleep duration >9 h/day showed no significant association (aPR = 1.13, 95% CI: 0.93–1.37, *p* = 0.232). Adequate physical activity (≥600 MET-min/week) was significantly associated with elevated depressive symptoms (aPR = 1.17, 95% CI: 1.04–1.32, *p* = 0.009). Drinking alcohol once or more per month was significantly associated with a lower prevalence of depressive symptoms (aPR = 0.86, 95% CI: 0.77–0.95, *p* = 0.004), whereas drinking less than once per month showed no significant association (aPR = 1.06, 95% CI: 0.90–1.25, *p* = 0.503). Smoking was not significantly associated with depressive symptoms (aPR = 1.05, 95% CI: 0.91–1.22, *p*= 0.490). Regarding social participation, engagement in leisure and recreational activities was significantly associated with a reduced prevalence of elevated depressive symptoms (aPR = 0.86, 95% CI: 0.77–0.96, *p* = 0.008), whereas no significant association was observed with social interactions with family or friends (aPR = 1.01, 95% CI: 0.93–1.08, *p* = 0.897), community activities (aPR = 0.92, 95% CI: 0.69–1.22, *p*= 0.556), volunteer/charitable activities (aPR = 0.96, 95% CI: 0.80–1.16, *p* = 0.688), or participation in training/learning activities (aPR = 0.88, 95% CI: 0.64–1.23, *p* = 0.462). Internet use showed no significant association with depressive symptoms (aPR = 0.98, 95% CI: 0.90–1.08, *p*= 0.712). Regarding demographic factors, males exhibited a significant association with lower prevalence of elevated depressive symptoms (aPR = 0.77, 95% CI: 0.70–0.86, *p* < 0.001). Compared to the 45–59 age group, individuals aged 75 years and above showed a significantly lower prevalence of elevated depressive symptoms (aPR = 0.75, 95% CI: 0.58–0.96, *p* = 0.022), while no significant difference was observed in the 60–74 age group (aPR = 0.87, 95% CI: 0.75–1.00, *p* = 0.059). The highest-income group demonstrated a markedly lower prevalence of elevated depressive symptoms compared to the lowest-income group (aPR = 0.60, 95% CI: 0.46–0.79, *p* < 0.001). In terms of health status variables, the number of chronic diseases (aPR = 1.10, 95% CI: 1.08–1.13, *p* < 0.001), the number of limitations in Activities of Daily Living (ADL) (aPR = 1.09, 95% CI: 1.06–1.13, *p* < 0.001), and the number of limitations in Instrumental Activities of Daily Living (IADL) (aPR = 1.14, 95% CI: 1.11–1.18, *p* < 0.001) were significantly associated with an elevated prevalence of depressive symptoms, whereas cognitive function scores were significantly linked to reduced elevated depressive symptoms (aPR = 0.97, 95% CI: 0.96–0.98, *p* < 0.001).

### 3.4. Ordinal Logistic Regression Analysis of Life Satisfaction

Table 7 presents the ordinal logistic regression results for life satisfaction, where an OR > 1 indicates an increased probability of higher life satisfaction. Sleep duration <7 h/day was significantly associated with lower life satisfaction (OR = 0.73, 95% CI: 0.67–0.79, *p* < 0.001), whereas sleep duration >9 h/day showed no significant association (OR = 1.16, 95% CI: 1.00–1.34, *p*= 0.051). Regarding social participation, volunteer/charitable activities were significantly associated with higher life satisfaction (OR = 1.43, 95% CI: 1.09–1.87, *p* = 0.010), while leisure and recreational activities (OR = 1.06, 95% CI: 0.95–1.18, *p* = 0.290), social interactions with family and friends (OR = 1.01, 95% CI: 0.92–1.11, *p* = 0.886), and other forms of social engagement showed no significant association. Internet use was significantly associated with lower life satisfaction (OR = 0.90, 95% CI: 0.81–1.00, *p* = 0.045). Among demographic factors, the 60–74-year-old group (OR = 1.13, 95% CI: 1.01–1.26, *p* = 0.029) and the 75-year-old and older group (OR = 1.42, 95% CI: 1.17–1.74, *p* < 0.001) exhibited higher life satisfaction compared to the 45–59-year-old group. Higher income was significantly associated with greater life satisfaction (highest-income group: OR = 1.34, 95% CI: 1.14–1.58, *p* < 0.001). Regarding health status variables, the number of chronic diseases (OR = 0.92, 95% CI: 0.90–0.94, *p* < 0.001), the number of limitations in ADLs (OR = 0.83, 95% CI: 0.78–0.88, *p* < 0.001), and the number of limitations in IADLs (OR = 0.86, 95% CI: 0.82–0.91, *p* < 0.001) were all significantly correlated with lower life satisfaction.

### 3.5. Subgroup Analysis and Interaction Testing

Subgroup analyses by sex demonstrated (Table 8) that the association between sleep deprivation (<7 h/day) and elevated depressive symptoms was significant in both women (aPR = 1.48, 95% CI: 1.31–1.67) and men (aPR = 1.50, 95% CI: 1.28–1.76), with similar effect sizes. The association between adequate physical activity and elevated depressive symptoms was only significant in women (aPR = 1.16, 95% CI: 1.03–1.31, *p* = 0.014) and not significant in men (aPR = 1.16, 95% CI: 0.94–1.44, *p* = 0.156). The association between leisure activities and reduced depressive symptoms was significant only in women (aPR = 0.82, 95% CI: 0.72–0.93, *p* = 0.002) and not significant in men (aPR = 0.92, 95% CI: 0.76–1.12, *p* = 0.416).

Subgroup analyses stratified by age group revealed (Table 9) that the association between insufficient sleep and elevated depressive symptoms was significant across all three age groups (45–59 years: aPR = 1.63, 95% CI: 1.36–1.95; 60–74 years: aPR = 1.37, 95% CI: 1.23–1.54; ≥75 years: aPR = 1.47, 95% CI: 1.08–2.00). The association between adequate physical activity and elevated depressive symptoms was significant in both the 45–59-year-old group (aPR = 1.25, 95% CI: 1.01–1.56, *p* = 0.039) and the ≥75-year-old group (aPR = 1.68, 95% CI: 1.20–2.36, *p* = 0.003), but not significant in the 60–74-year-old group (aPR = 1.03, 95% CI: 0.90–1.18, *p* = 0.678).

The interaction analysis (Table 10) revealed significant interactions between sex and adequate physical activity and life satisfaction (interaction *p* = 0.025); between age group and adequate physical activity and depressive symptoms (interaction *p* = 0.025); between age group and community engagement and depressive symptoms (interaction *p* = 0.047); between sex and internet use and life satisfaction (interaction *p* = 0.012); and between age group and internet use and life satisfaction (interaction *p* = 0.007). No statistically significant interactions were observed between other exposure variables and either sex or age group.

### 3.6. Sensitivity Analysis

To evaluate the robustness of covariate adjustment within the DAG framework, we compared the “base adjustment model” incorporating only demographic and socioeconomic covariates with the “full adjustment model” that additionally included health status variables (number of chronic diseases, ADL, IADL, cognitive function) (Supplementary Table S1). The results demonstrated that the association between sleep deprivation and depressive symptoms was stronger in the base model (aPR = 1.69, 95% CI: 1.51–1.89) than in the full model (aPR = 1.49, 95% CI: 1.33–1.68), indicating that the association was attenuated after adjusting for health status variables. The association between leisure activities and lower prevalence of depressive symptoms remained significant in both the base model (aPR = 0.83, 95% CI: 0.74–0.92) and the full model (aPR = 0.86, 95% CI: 0.77–0.96), with stable effect estimates. The association between alcohol consumption (once or more per month) and depressive symptoms was stronger in the base model (aPR = 0.80, 95% CI: 0.72–0.89) and weakened but remained significant in the full model (aPR = 0.86, 95% CI: 0.77–0.95), indicating that the association was attenuated after adjusting for health status variables.

The separate modeling analysis of digital behaviors (Supplementary Table S2) demonstrated that, after adjusting for all covariates, watching videos (aPR = 0.87, 95% CI: 0.76–0.99, *p* = 0.042) and playing online games (aPR = 0.65, 95% CI: 0.50–0.83, *p* < 0.001) were significantly associated with reduced depressive symptoms; other digital behaviors (chatting, reading news, mobile payments, WeChat usage, and posting on Moments) showed no significant association with depressive symptoms. Regarding life satisfaction, playing online games was significantly correlated with higher life satisfaction (OR = 1.29, 95% CI: 1.01–1.64, *p* = 0.043), whereas mobile payments (OR = 0.75, 95% CI: 0.65–0.87, *p* < 0.001), WeChat usage (OR = 0.80, 95% CI: 0.65–0.99, *p* = 0.043), and internet use (OR = 0.90, 95% CI: 0.81–1.00, *p* = 0.045) were significantly associated with lower life satisfaction.

In the sensitivity analysis of sleep duration (Supplementary Table S3), using the same three-category sleep duration classification with a reduced sample (*n* = 13,490) after excluding participants with missing covariate data, the results were consistent with the main model’s findings. Multiple imputation for missing data yielded results consistent with the complete-case analysis. Details on missing data characteristics are presented in Supplementary Table S4.

## 4. Discussion

This study utilized data from the 2020 CHARLS national representative survey to analyze the associations between sleep duration, physical activity, digital engagement, and social participation and depressive symptoms and life satisfaction among middle-aged and older adults. The findings revealed that insufficient sleep (<7 h/day) was significantly associated with increased depressive symptoms and reduced life satisfaction; participation in leisure and recreational activities was significantly associated with decreased depressive symptoms; watching videos and playing online games were significantly associated with reduced depressive symptoms; and overall use of mobile payments, WeChat, and the internet was associated with lower life satisfaction. Furthermore, adequate physical activity was associated with a higher prevalence of elevated depressive symptoms—a finding consistent with several recent CHARLS-based studies (R. Wang et al., 2025), which may be attributed to the fact that CHARLS’s measurement of physical activity included a wide range of non-leisure activities (e.g., farming, household chores). Additionally, women, rural residents, and low-income groups exhibited higher prevalence of elevated depressive symptoms. These findings suggest that behavioral intervention strategies targeting sleep hygiene, leisure activity participation, and differentiated digital behaviors warrant further attention. Given that the CHARLS 2020 data were collected during the COVID-19 pandemic, the observed associations may have been influenced by pandemic-related changes in mental health and health behaviors. The cross-sectional design prevents us from quantifying this potential impact.

The weighted prevalence of depressive symptoms in this study was 44.5%, consistent with the previous literature (Hu et al., 2024; X. Zhao et al., 2025). Women, rural residents, and low-income groups exhibited significantly higher prevalence of elevated depressive symptoms, highlighting the significant role of sociodemographic factors in mental health disparities (Hu et al., 2024; X. Zhao et al., 2025). The association between sleep deprivation and elevated depressive symptoms as well as reduced life satisfaction was the most robust finding in this study, aligning with prior research (Hu et al., 2024; X. Li et al., 2025; R. Wang et al., 2025; X. Zhao et al., 2025; Y. Zhao et al., 2023). The categorical analysis did not provide clear evidence that both short and long sleep durations were associated with depressive symptoms.

Adequate physical activity was associated with a higher prevalence of elevated depressive symptoms (aPR = 1.17, *p* = 0.009), a finding consistent with several recent studies based on the CHARLS scale (S. Li et al., 2024; R. Wang et al., 2025). The CHARLS physical activity measurement covers three dimensions: work-related activities (including agricultural tasks), transportation, and leisure exercise; non-leisure physical activity constitutes a significant proportion of this sample (46.8% among rural residents). In subgroup analyses, this association was only significant among women (aPR = 1.16, *p* = 0.014), aligning with the previous literature. These results suggest that when developing physical activity intervention strategies for middle-aged and older adults, it is essential to distinguish between leisure and non-leisure activities and account for urban–rural and sex differences.

In terms of social participation, leisure and recreational activities were significantly associated with reduced depressive symptoms (aPR = 0.86, *p* = 0.008), consistent with previous studies (X. Zhang et al., 2024). No significant association was observedbetween social interactions and family/friends, community activities, volunteer/charitable work, or training/learning activities. In subgroup analyses, this negative correlation was only statistically significant among women (aPR = 0.82, *p* = 0.002). Regarding life satisfaction, volunteer/charitable activities showed a significant positive correlation (OR = 1.43, *p* = 0.010). Overall internet usage exhibited no significant association with depressive symptoms (aPR = 0.98, *p* = 0.712) but was significantly linked to lower life satisfaction (OR= 0.90, *p* = 0.045). Separate analyses of digital behaviors revealed that video watching and online gaming were significantly associated with reduced depressive symptoms, whereas chatting, news consumption, mobile payments, and WeChat usage showed no significant associations (Qi et al., 2024). For life satisfaction, mobile payments and WeChat usage were significantly associated with lower levels. Interaction tests demonstrated significant interaction effects between sex and age groups and internet usage on life satisfaction.

The COVID-19 pandemic drastically altered daily activity routines, serving as a critical contextual background for findings from the 2020 CHARLS survey. Containment policies restricted offline social and recreational activities and disrupted interpersonal support networks. Previous research indicates such restrictions raise depressive risk and weaken the mental health benefits of in-person socialising (Du et al., 2022; Kohls et al., 2021). This loss of face-to-face contact may explain the stronger association between lower life satisfaction and elevated depressive symptoms observed in our sample, as older adults received less emotional support and belonging from offline interactions (Y. L. Chen et al., 2025; Y. P. Li et al., 2025). Meanwhile, older adults shifted toward home-based leisure and family activities, with leisure participation acting as a key mediator linking social media use and depressive symptoms (Maruta et al., 2023). Digital engagement partially offset limited offline contact, yet its benefits depended on usage patterns. Consistent with our results, older adults maintaining alternative leisure and online social activities exhibited better life satisfaction and milder depressive symptoms, whereas isolated individuals lacking recreational alternatives experienced greater psychological burden.

This study offers the following implications for public health practice: sleep hygiene should be prioritized as an intervention target; physical activity interventions require differentiation between recreational and non-recreational activities; recreational activities are cost-effective, easy to promote, and suitable for inclusion in community-based older adult service programs; and digital health interventions must account for the distinct effects of different types of activities.

This study has the following limitations. The cross-sectional design cannot establish causal relationships; although the present study incorporated health status variables (chronic diseases, ADL, IADL, cognitive function) as baseline characteristics within a DAG framework, the temporal ordering between these variables and the exposures cannot be established in cross-sectional data. All variables were self-reported; the CHARLS physical activity scale combines three types of activities to calculate MET values, failing to distinguish between the differential effects of different activity types; CES-D10 is a screening tool, and elevated depressive symptoms in this study do not equate to clinical depression; and potential mediating variables such as psychosocial resources were not included. Notably, our findings suffer from limited generalizability, as we solely utilized the 2020 CHARLS wave collected amid the COVID-19 pandemic. Pandemic restrictions altered respondents’ lifestyles and social patterns, so the observed associations only apply to the pandemic period and cannot be extended to pre- or post-pandemic middle-aged and older adults. Future research should employ a longitudinal design, differentiate between types of physical activity, explore quality dimensions of digital participation, and evaluate the efficacy of relevant mental health promotion interventions through randomized controlled trials.

## 5. Conclusions

This study analyzed the associations between sleep duration, physical activity, digital participation, social engagement, and depressive symptoms as well as life satisfaction among middle-aged and older adults in China based on data from CHARLS 2020. The weighted prevalence of depressive symptoms was 44.5%. The association between sleep deprivation and elevated depressive symptoms as well as reduced life satisfaction was the most robust; participation in leisure and recreational activities was significantly associated with decreased depressive symptoms; adequate physical activity was associated with a higher prevalence of elevated depressive symptoms; and watching videos and playing online games were significantly associated with reduced depressive symptoms, whereas overall internet use was associated with lower life satisfaction. Women, rural residents, and low-income groups had a higher prevalence of elevated depressive symptoms. This study employed a cross-sectional design, and causal inference requires further validation through longitudinal research.

## Figures and Tables

**Table 1:** Sample selection flow for the CHARLS 2020 analysis.

Analysis Path	Step	Eligibility Criterion	n
Overall	0	Merged CHARLS 2020	19,395
Overall	1	Age ≥ 45	19,157
Overall	2	Valid weight and PSU	17,232
Elevated depressive symptoms	3	Nonmissing outcome	14,419
Elevated depressive symptoms	4	Complete model sample	13,576
Life satisfaction	3	Nonmissing outcome	15,715
Life satisfaction	4	Complete model sample	14,530

PSU = primary sampling unit. No unofficial stratification variable was specified.

**Table 2:** DAG-informed covariate adjustment set.

DAG Role	Variables	Adjustment Assumption
Demographic causes	sex, age3	Common causes of behavior/social participation and both outcomes
Socioeconomic/contextual causes	income_q, region, married, Medical_Insurance, edu_3c, urban_nbs, household_menbers, alive_child	Pre-exposure determinants of access, participation and mental well-being
Baseline health/function/cognition	chronic_disease, r5adlab_c, r5iadl, cognition	Treated as pre-exposure common causes; cross-sectional temporality must be acknowledged

Health, functional and cognitive measures were treated as pre-exposure common causes in the full model. Because the data are cross-sectional, temporality cannot be established.

**Table 3:** Unweighted and survey-weighted characteristics of the elevated depressive symptoms analysis sample (*n* = 13,576).

Characteristic	Unweighted, *n* (%) or Mean (SD)	Survey-Weighted, % or Mean (SD)
Age	60.995 (8.840)	61.293 (9.509)
Income	13,579.271 (23,579.219)	20,379.268 (42,914.052)
Household members	1.109 (1.545)	1.117 (1.594)
Number of living children	2.418 (1.214)	2.322 (1.253)
Number of chronic diseases	2.299 (1.962)	2.282 (1.970)
ADL limitations	0.389 (0.959)	0.359 (0.930)
IADL limitations	0.426 (1.027)	0.396 (1.004)
Core cognition score	11.561 (4.310)	11.973 (4.381)
Sleep duration	406.083 (121.688)	405.168 (119.367)
Physical activity (MET-min/week)	6078.846 (5910.553)	5635.047 (5659.488)
CESD	6.293 (5.805)	5.976 (5.671)
Sex		
Female	6950 (51.2%)	50.2%
Male	6626 (48.8%)	49.8%
Age group		
45–59	6308 (46.5%)	46.6%
60–74	6199 (45.7%)	42.8%
75+	1069 (7.9%)	10.6%
Income quintile		
Q1 (lowest income)	3687 (27.2%)	24.9%
Q2 (lower-middle income)	2345 (17.3%)	14.3%
Q3 (middle income)	2929 (21.6%)	18.7%
Q4 (upper-middle income)	2719 (20.0%)	21.0%
Q5 (highest income)	1896 (14.0%)	21.2%
Region		
Northeast region	914 (6.7%)	9.0%
Eastern region	4255 (31.3%)	35.1%
Central region	3959 (29.2%)	25.5%
Western region	4448 (32.8%)	30.5%
Marital status		
Single	1843 (13.6%)	14.0%
Married	11,733 (86.4%)	86.0%
Medical insurance		
Not participating in medical insurance	536 (3.9%)	3.9%
Residential medical insurance	11,023 (81.2%)	72.1%
Enterprise employee medical insurance	1868 (13.8%)	22.4%
Public medical care	149 (1.1%)	1.6%
Education level		
Education—Primary and lower	8435 (62.1%)	55.7%
Education—Secondary school	4854 (35.8%)	40.5%
Education—Junior college and above	287 (2.1%)	3.8%
Residence		
Rural	8318 (61.3%)	46.8%
Urban	5258 (38.7%)	53.2%
Total daily sleep duration		
<7 h	6490 (47.8%)	48.6%
7–9 h	5861 (43.2%)	43.0%
>9 h	1225 (9.0%)	8.4%
Physical activity sufficiency		
<600 MET-min/week	1936 (14.3%)	13.8%
≥600 MET-min/week	11,640 (85.7%)	86.2%
Smoking status		
Non-smoker	9949 (73.3%)	73.9%
Smoker	3627 (26.7%)	26.1%
Alcohol consumption status		
Does not drink alcohol	8422 (62.0%)	60.2%
Drinks alcohol—less than once a month	1352 (10.0%)	10.7%
Drinks alcohol—more than once a month	3802 (28.0%)	29.1%
Participates in social activities with friends and relatives		
No	8213 (60.5%)	59.5%
Yes	5363 (39.5%)	40.5%
Participates in recreational and leisure social activities		
No	10,637 (78.4%)	77.3%
Yes	2939 (21.6%)	22.7%
Participates in community activities		
No	13,212 (97.3%)	96.3%
Yes	364 (2.7%)	3.7%
Participates in volunteer/charity activities		
No	13,111 (96.6%)	96.1%
Yes	465 (3.4%)	3.9%
Participates in school/training activities		
No	13,353 (98.4%)	98.1%
Yes	223 (1.6%)	1.9%
Internet usage status		
Does not use the Internet	7657 (56.4%)	51.4%
Uses the Internet	5919 (43.6%)	48.6%

Unweighted categorical summaries are n (%). Survey-weighted categorical summaries are weighted percentages; continuous variables are mean (SD).

**Table 4:** Unweighted and survey-weighted characteristics of the life-satisfaction analysis sample (n = 14,530).

Characteristic	Unweighted, *n* (%) or Mean (SD)	Survey-Weighted, % or Mean (SD)
Age	61.418 (9.024)	61.720 (9.740)
Income	13,135.275 (23,154.977)	19,713.921 (42,076.050)
Household members	1.111 (1.554)	1.119 (1.603)
Number of living children	2.461 (1.243)	2.366 (1.285)
Number of chronic diseases	2.316 (1.969)	2.300 (1.984)
ADL limitations	0.413 (0.994)	0.381 (0.967)
IADL limitations	0.461 (1.073)	0.433 (1.060)
Core cognition score	11.277 (4.463)	11.692 (4.564)
Sleep duration	405.742 (123.870)	404.947 (122.266)
Physical activity (MET-min/week)	6054.529 (5921.151)	5613.430 (5693.600)
Life satisfaction (0–4)	2.270 (0.781)	2.265 (0.772)
Sex		
Female	7574 (52.1%)	51.1%
Male	6956 (47.9%)	48.9%
Age group		
45–59	6510 (44.8%)	45.0%
60–74	6719 (46.2%)	43.2%
75+	1301 (9.0%)	11.8%
Income quintile		
Q1 (lowest income)	3913 (26.9%)	24.7%
Q2 (lower-middle income)	2603 (17.9%)	14.9%
Q3 (middle income)	3227 (22.2%)	19.3%
Q4 (upper-middle income)	2832 (19.5%)	20.5%
Q5 (highest income)	1955 (13.5%)	20.6%
Region		
Northeast region	923 (6.4%)	8.5%
Eastern region	4563 (31.4%)	35.0%
Central region	4259 (29.3%)	25.7%
Western region	4785 (32.9%)	30.7%
Marital status		
Single	2104 (14.5%)	15.0%
Married	12,426 (85.5%)	85.0%
Medical insurance		
Not participating in medical insurance	597 (4.1%)	4.0%
Residential medical insurance	11,850 (81.6%)	72.7%
Enterprise employee medical insurance	1927 (13.3%)	21.7%
Public medical care	156 (1.1%)	1.6%
Education level		
Education—Primary and lower	9298 (64.0%)	57.7%
Education—Secondary school	4943 (34.0%)	38.8%
Education—Junior college and above	289 (2.0%)	3.6%
Residence		
Rural	8990 (61.9%)	47.6%
Urban	5540 (38.1%)	52.4%
Total daily sleep duration		
<7 h	6966 (47.9%)	48.6%
7–9 h	6200 (42.7%)	42.5%
>9 h	1364 (9.4%)	8.8%
Physical activity sufficiency		
<600 MET-min/week	2150 (14.8%)	14.5%
≥600 MET-min/week	12,380 (85.2%)	85.5%
Smoking status		
Non-smoker	10,710 (73.7%)	74.1%
Smoker	3820 (26.3%)	25.9%
Alcohol consumption status		
Does not drink alcohol	9156 (63.0%)	61.1%
Drinks alcohol—less than once a month	1408 (9.7%)	10.5%
Drinks alcohol—more than once a month	3966 (27.3%)	28.4%
Participates in social activities with friends and relatives		
No	8842 (60.9%)	59.9%
Yes	5688 (39.1%)	40.1%
Participates in recreational and leisure social activities		
No	11,459 (78.9%)	77.8%
Yes	3071 (21.1%)	22.2%
Participates in community activities		
No	14,157 (97.4%)	96.4%
Yes	373 (2.6%)	3.6%
Participates in volunteer/charity activities		
No	14,047 (96.7%)	96.2%
Yes	483 (3.3%)	3.8%
Participates in school/training activities		
No	14,300 (98.4%)	98.1%
Yes	230 (1.6%)	1.9%
Internet usage status		
Does not use the Internet	8486 (58.4%)	53.4%
Uses the Internet	6044 (41.6%)	46.6%

Unweighted categorical summaries are n (%). Survey-weighted categorical summaries are weighted percentages; continuous variables are mean (SD). Income quintiles were based on weighted individual-income quintiles using survey weights. Cutoffs (in 2020 CNY): Q1 (lowest): ≤¥0; Q2 (lower-middle): >¥0–¥1320; Q3 (middle): >¥1320–¥10,000; Q4 (upper-middle): >¥10,000–¥35,420; Q5 (highest): >¥35,420.

**Table 5:** Unweighted and survey-weighted univariable association tests.

Outcome	Factor	Unweighted F	*p*-Value	Survey-Weighted F	*p*-Value
Elevated depressive symptoms	Sex	363.05	<0.001	259.60	<0.001
Elevated depressive symptoms	Age group	13.86	<0.001	1.65	0.193
Elevated depressive symptoms	Income quintile	134.65	<0.001	68.64	<0.001
Elevated depressive symptoms	Region	57.83	<0.001	12.58	<0.001
Elevated depressive symptoms	Marital status	107.40	<0.001	47.17	<0.001
Elevated depressive symptoms	Medical insurance	70.96	<0.001	50.47	<0.001
Elevated depressive symptoms	Education level	168.48	<0.001	92.52	<0.001
Elevated depressive symptoms	Residence	181.36	<0.001	64.39	<0.001
Elevated depressive symptoms	Sleep-duration category	215.05	<0.001	74.83	<0.001
Elevated depressive symptoms	Physical activity sufficiency	12.73	<0.001	5.26	0.022
Elevated depressive symptoms	Smoking status	82.06	<0.001	36.09	<0.001
Elevated depressive symptoms	Alcohol consumption status	102.23	<0.001	75.11	<0.001
Elevated depressive symptoms	Social activities with friends/relatives	5.05	0.025	8.77	0.003
Elevated depressive symptoms	Recreational/leisure activities	39.45	<0.001	37.92	<0.001
Elevated depressive symptoms	Community activities	11.46	<0.001	13.71	<0.001
Elevated depressive symptoms	Volunteer/charity activities	2.48	0.115	12.86	<0.001
Elevated depressive symptoms	School/training activities	5.90	0.015	18.82	<0.001
Elevated depressive symptoms	Internet use	149.94	<0.001	109.34	<0.001
Life satisfaction	Sex	10.52	0.001	1.77	0.184
Life satisfaction	Age group	16.07	<0.001	9.03	<0.001
Life satisfaction	Income quintile	9.69	<0.001	4.24	0.002
Life satisfaction	Region	2.11	0.096	0.47	0.707
Life satisfaction	Marital status	11.49	<0.001	2.26	0.134
Life satisfaction	Medical insurance	6.70	<0.001	7.27	<0.001
Life satisfaction	Education level	3.50	0.030	5.36	0.005
Life satisfaction	Residence	0.88	0.348	0.11	0.743
Life satisfaction	Sleep-duration category	84.85	<0.001	41.27	<0.001
Life satisfaction	Physical activity sufficiency	3.74	0.053	2.61	0.107
Life satisfaction	Smoking status	1.10	0.295	0.88	0.347
Life satisfaction	Alcohol consumption status	6.43	0.002	3.33	0.037
Life satisfaction	Social activities with friends/relatives	0.32	0.573	0.13	0.714
Life satisfaction	Recreational/leisure activities	7.00	0.008	1.21	0.272
Life satisfaction	Community activities	2.88	0.090	0.06	0.812
Life satisfaction	Volunteer/charity activities	2.87	0.090	3.85	0.051
Life satisfaction	School/training activities	0.03	0.872	0.55	0.460
Life satisfaction	Internet use	8.26	0.004	4.27	0.039

Survey-weighted tests account for individual weights and PSU clustering. *p*-values are two-sided.

**Table 6:** Modified Poisson regression for elevated depressive symptoms.

Variable	Level	Unweighted Model		Survey-Weighted Model	
		PR (95% CI)	*p* Value	PR (95% CI)	*p* Value
Total daily sleep duration	7–9 h	1.00 (Reference)		1.00 (Reference)	
	<7 h	1.46 (1.36–1.55)	<0.001	1.49 (1.33–1.68)	<0.001
	>9 h	0.98 (0.87–1.09)	0.673	1.13 (0.93–1.37)	0.232
Physical activity sufficiency	<600 MET-min/week	1.00 (Reference)		1.00 (Reference)	
	≥600 MET-min/week	1.17 (1.08–1.26)	<0.001	1.17 (1.04–1.32)	0.009
Smoking status	Non-smoker	1.00 (Reference)		1.00 (Reference)	
	Smoker	1.09 (1.00–1.18)	0.050	1.05 (0.91–1.22)	0.490
Alcohol consumption status	Does not drink alcohol	1.00 (Reference)		1.00 (Reference)	
	Drinks alcohol—less than once a month	1.03 (0.93–1.13)	0.590	1.06 (0.90–1.25)	0.503
	Drinks alcohol—more than once a month	0.89 (0.82–0.96)	0.004	0.86 (0.77–0.95)	0.004
Participates in social activities with friends and relatives	No	1.00 (Reference)		1.00 (Reference)	
	Yes	1.02 (0.97–1.08)	0.397	1.01 (0.93–1.08)	0.897
Participates in recreational and leisure social activities	No	1.00 (Reference)		1.00 (Reference)	
	Yes	0.92 (0.85–0.99)	0.025	0.86 (0.77–0.96)	0.008
Participates in community activities	No	1.00 (Reference)		1.00 (Reference)	
	Yes	0.95 (0.77–1.16)	0.596	0.92 (0.69–1.22)	0.556
Participates in volunteer/charity activities	No	1.00 (Reference)		1.00 (Reference)	
	Yes	1.08 (0.93–1.26)	0.303	0.96 (0.80–1.16)	0.688
Participates in school/training activities	No	1.00 (Reference)		1.00 (Reference)	
	Yes	1.12 (0.85–1.48)	0.430	0.88 (0.64–1.23)	0.462
Internet usage status	Does not use the Internet	1.00 (Reference)		1.00 (Reference)	
	Uses the Internet	0.97 (0.91–1.04)	0.442	0.98 (0.90–1.08)	0.712
Sex	Female	1.00 (Reference)		1.00 (Reference)	
	Male	0.74 (0.69–0.80)	<0.001	0.77 (0.70–0.86)	<0.001
Age group	45–59	1.00 (Reference)		1.00 (Reference)	
	60–74	0.89 (0.82–0.97)	0.008	0.87 (0.75–1.00)	0.059
	75+	0.73 (0.64–0.84)	<0.001	0.75 (0.58–0.96)	0.022
Income quintile	Lowest-income group	1.00 (Reference)		1.00 (Reference)	
	Q2 (lower-middle income)	1.00 (0.92–1.08)	0.947	0.98 (0.87–1.09)	0.664
	Q3 (middle income)	1.00 (0.92–1.09)	0.965	1.02 (0.91–1.15)	0.682
	Q4 (upper-middle income)	0.79 (0.72–0.87)	<0.001	0.77 (0.64–0.93)	0.006
	Q5 (highest income)	0.58 (0.49–0.69)	<0.001	0.60 (0.46–0.79)	<0.001
Region	Northeast region	1.00 (Reference)		1.00 (Reference)	
	Eastern region	0.98 (0.86–1.13)	0.809	1.07 (0.87–1.33)	0.507
	Central region	1.31 (1.16–1.49)	<0.001	1.34 (1.15–1.56)	<0.001
	Western region	1.21 (1.06–1.39)	0.005	1.24 (1.06–1.46)	0.008
Marital status	Not married	1.00 (Reference)		1.00 (Reference)	
	Married	0.88 (0.81–0.94)	<0.001	0.91 (0.83–0.99)	0.030
Medical insurance	Not participating in medical insurance	1.00 (Reference)		1.00 (Reference)	
	Residential medical insurance	0.89 (0.79–1.01)	0.081	0.92 (0.78–1.08)	0.296
	Enterprise employee medical insurance	0.86 (0.72–1.02)	0.090	0.87 (0.66–1.14)	0.313
	Public medical care	0.79 (0.47–1.33)	0.383	0.67 (0.38–1.21)	0.187
Education level	Primary school and lower	1.00 (Reference)		1.00 (Reference)	
	Education—Secondary school	0.88 (0.81–0.95)	0.002	0.91 (0.77–1.07)	0.269
	Education—Junior college and above	0.69 (0.48–0.97)	0.034	0.63 (0.40–0.99)	0.044
Residence	Rural	1.00 (Reference)		1.00 (Reference)	
	Urban	0.86 (0.78–0.94)	0.002	0.89 (0.78–1.01)	0.065
Household members	Per 1-unit increase	0.99 (0.97–1.01)	0.349	0.98 (0.96–1.01)	0.139
Number of living children	Per 1-unit increase	1.00 (0.97–1.03)	0.933	1.01 (0.97–1.04)	0.743
Number of chronic diseases	Per 1-unit increase	1.11 (1.10–1.13)	<0.001	1.10 (1.08–1.13)	<0.001
ADL limitations	Per 1-unit increase	1.09 (1.07–1.12)	<0.001	1.09 (1.06–1.13)	<0.001
IADL limitations	Per 1-unit increase	1.12 (1.09–1.14)	<0.001	1.14 (1.11–1.18)	<0.001
Core cognition score	Per 1-unit increase	0.97 (0.96–0.98)	<0.001	0.97 (0.96–0.98)	<0.001

Values are prevalence ratios (PRs) with 95% CIs. Both models include all displayed exposures and DAG-informed covariates. The weighted model accounts for individual weights and PSU clustering. Income quintiles were based on weighted individual-income quintiles using survey weights. Cutoffs (in 2020 CNY): Q1 (lowest): ≤¥0; Q2 (lower-middle): >¥0–¥1320; Q3 (middle): >¥1320–¥10,000; Q4 (upper-middle): >¥10,000–¥35,420; Q5 (highest): >¥35,420.

**Table 7:** Ordinal logistic regression for life satisfaction.

Variable	Level	Unweighted Model		Survey-Weighted Model	
		OR (95% CI)	*p* Value	OR (95% CI)	*p* Value
Total daily sleep duration	7–9 h	1.00 (Reference)		1.00 (Reference)	
	<7 h	0.76 (0.71–0.81)	<0.001	0.73 (0.67–0.79)	<0.001
	>9 h	1.17 (1.04–1.32)	0.009	1.16 (1.00–1.34)	0.051
Physical activity sufficiency	<600 MET-min/week	1.00 (Reference)		1.00 (Reference)	
	≥600 MET-min/week	0.94 (0.85–1.05)	0.302	0.96 (0.81–1.15)	0.667
Smoking status	Non-smoker	1.00 (Reference)		1.00 (Reference)	
	Smoker	0.93 (0.85–1.01)	0.077	0.97 (0.85–1.10)	0.605
Alcohol consumption status	Does not drink alcohol	1.00 (Reference)		1.00 (Reference)	
	Drinks alcohol—less than once a month	0.89 (0.79–1.00)	0.045	0.88 (0.75–1.03)	0.105
	Drinks alcohol—more than once a month	1.03 (0.94–1.12)	0.576	1.05 (0.94–1.17)	0.368
Participates in social activities with friends and relatives	No	1.00 (Reference)		1.00 (Reference)	
	Yes	0.99 (0.93–1.07)	0.849	1.01 (0.92–1.11)	0.886
Participates in recreational and leisure social activities	No	1.00 (Reference)		1.00 (Reference)	
	Yes	1.10 (1.01–1.19)	0.020	1.06 (0.95–1.18)	0.290
Participates in community activities	No	1.00 (Reference)		1.00 (Reference)	
	Yes	1.11 (0.91–1.35)	0.325	0.80 (0.47–1.37)	0.418
Participates in volunteer/charity activities	No	1.00 (Reference)		1.00 (Reference)	
	Yes	1.21 (1.01–1.45)	0.038	1.43 (1.09–1.87)	0.010
Participates in school/training activities	No	1.00 (Reference)		1.00 (Reference)	
	Yes	1.03 (0.78–1.34)	0.854	0.81 (0.41–1.61)	0.552
Internet usage status	Does not use the Internet	1.00 (Reference)		1.00 (Reference)	
	Uses the Internet	0.87 (0.80–0.94)	<0.001	0.90 (0.81–1.00)	0.045
Sex	Female	1.00 (Reference)		1.00 (Reference)	
	Male	0.95 (0.87–1.03)	0.196	0.89 (0.79–1.00)	0.058
Age group	45–59	1.00 (Reference)		1.00 (Reference)	
	60–74	1.14 (1.05–1.25)	0.003	1.13 (1.01–1.26)	0.029
	75+	1.58 (1.35–1.84)	<0.001	1.42 (1.17–1.74)	<0.001
Income quintile	Lowest-income group	1.00 (Reference)		1.00 (Reference)	
	Q2 (lower-middle income)	1.17 (1.04–1.31)	0.009	1.14 (0.98–1.32)	0.084
	Q3 (middle income)	1.14 (1.02–1.27)	0.016	1.18 (1.03–1.34)	0.016
	Q4 (upper-middle income)	1.14 (1.02–1.27)	0.024	1.16 (1.01–1.33)	0.036
	Q5 (highest income)	1.28 (1.12–1.46)	<0.001	1.34 (1.14–1.58)	<0.001
Region	Northeast region	1.00 (Reference)		1.00 (Reference)	
	Eastern region	0.89 (0.76–1.05)	0.179	0.78 (0.63–0.96)	0.019
	Central region	0.88 (0.75–1.03)	0.116	0.79 (0.65–0.96)	0.015
	Western region	0.89 (0.76–1.06)	0.186	0.82 (0.67–1.00)	0.051
Marital status	Not married	1.00 (Reference)		1.00 (Reference)	
	Married	1.13 (1.02–1.27)	0.024	1.09 (0.95–1.25)	0.199
Medical insurance	Not participating in medical insurance	1.00 (Reference)		1.00 (Reference)	
	Residential medical insurance	1.15 (0.94–1.42)	0.172	1.33 (1.05–1.68)	0.018
	Enterprise employee medical insurance	1.11 (0.88–1.39)	0.376	1.43 (1.09–1.88)	0.009
	Public medical care	1.80 (1.21–2.67)	0.003	2.37 (1.29–4.37)	0.006
Education level	Primary school and lower	1.00 (Reference)		1.00 (Reference)	
	Education—Secondary school	0.81 (0.75–0.88)	<0.001	0.79 (0.70–0.88)	<0.001
	Education—Junior college and above	0.87 (0.69–1.09)	0.221	0.88 (0.61–1.27)	0.494
Residence	Rural	1.00 (Reference)		1.00 (Reference)	
	Urban	0.99 (0.89–1.10)	0.849	0.91 (0.80–1.04)	0.165
Household members	Per 1-unit increase	1.04 (1.01–1.06)	0.002	1.07 (1.03–1.10)	<0.001
Number of living children	Per 1-unit increase	1.04 (1.01–1.08)	0.019	1.07 (1.03–1.11)	0.002
Number of chronic diseases	Per 1-unit increase	0.91 (0.89–0.93)	<0.001	0.92 (0.90–0.94)	<0.001
ADL limitations	Per 1-unit increase	0.85 (0.81–0.89)	<0.001	0.83 (0.78–0.88)	<0.001
IADL limitations	Per 1-unit increase	0.86 (0.83–0.90)	<0.001	0.86 (0.82–0.91)	<0.001
Core cognition score	Per 1-unit increase	1.00 (0.99–1.01)	0.777	0.99 (0.98–1.01)	0.319

Values are odds ratios (ORs) with 95% CIs; OR >1 indicates higher odds of greater life satisfaction. Both models include all displayed exposures and DAG-informed covariates. Income quintiles were based on weighted individual-income quintiles using survey weights. Cutoffs (in 2020 CNY): Q1 (lowest): ≤¥0; Q2 (lower-middle): >¥0–¥1320; Q3 (middle): >¥1320–¥10,000; Q4 (upper-middle): >¥10,000–¥35,420; Q5 (highest): >¥35,420.

**Table 8:** Sex-specific associations with elevated depressive symptoms and life satisfaction.

**Panel A. Elevated Depressive Symptoms**					
**Variable**	**Level**	**Female**		**Male**	
		**PR (95% CI)**	***p* Value**	**PR (95% CI)**	***p* Value**
Total daily sleep duration	7–9 h	1.00 (Reference)		1.00 (Reference)	
	<7 h	1.48 (1.31–1.67)	<0.001	1.50 (1.28–1.76)	<0.001
	>9 h	1.07 (0.82–1.40)	0.600	1.17 (0.83–1.64)	0.379
Physical activity sufficiency	<600 MET-min/week	1.00 (Reference)		1.00 (Reference)	
	≥600 MET-min/week	1.16 (1.03–1.31)	0.014	1.16 (0.94–1.44)	0.156
Smoking status	Non-smoker	1.00 (Reference)		1.00 (Reference)	
	Smoker	1.10 (0.90–1.35)	0.338	1.06 (0.89–1.27)	0.518
Alcohol consumption status	Does not drink alcohol	1.00 (Reference)		1.00 (Reference)	
	Drinks alcohol—less than once a month	1.05 (0.87–1.28)	0.597	1.04 (0.79–1.36)	0.791
	Drinks alcohol—more than once a month	1.06 (0.87–1.30)	0.543	0.78 (0.63–0.97)	0.024
Participates in social activities with friends and relatives	No	1.00 (Reference)		1.00 (Reference)	
	Yes	1.02 (0.91–1.14)	0.772	1.00 (0.78–1.28)	0.986
Participates in recreational and leisure social activities	No	1.00 (Reference)		1.00 (Reference)	
	Yes	0.82 (0.72–0.93)	0.002	0.92 (0.76–1.12)	0.416
Participates in community activities	No	1.00 (Reference)		1.00 (Reference)	
	Yes	0.84 (0.55–1.29)	0.426	1.00 (0.69–1.44)	0.986
Participates in volunteer/charity activities	No	1.00 (Reference)		1.00 (Reference)	
	Yes	0.90 (0.69–1.17)	0.429	1.05 (0.78–1.42)	0.753
Participates in school/training activities	No	1.00 (Reference)		1.00 (Reference)	
	Yes	0.80 (0.50–1.27)	0.338	0.97 (0.61–1.55)	0.906
Internet usage status	Does not use the Internet	1.00 (Reference)		1.00 (Reference)	
	Uses the Internet	0.98 (0.87–1.11)	0.757	0.99 (0.80–1.21)	0.898
**Panel B. Life Satisfaction**					
**Variable**	**Level**	**Female**		**Male**	
		**OR (95% CI)**	***p* Value**	**OR (95% CI)**	***p* Value**
Total Daily Sleep Duration	7–9 h	1.00 (Reference)		1.00 (Reference)	
	<7 h	0.73 (0.65–0.81)	<0.001	0.72 (0.64–0.82)	<0.001
	>9 h	1.25 (1.00–1.55)	0.046	1.11 (0.94–1.31)	0.232
Physical activity sufficiency	<600 MET-min/week	1.00 (Reference)		1.00 (Reference)	
	≥600 MET-min/week	0.83 (0.70–1.00)	0.046	1.11 (0.85–1.45)	0.439
Smoking status	Non-smoker	1.00 (Reference)		1.00 (Reference)	
	Smoker	1.13 (0.84–1.53)	0.409	0.93 (0.81–1.06)	0.288
Alcohol consumption status	Does not drink alcohol	1.00 (Reference)		1.00 (Reference)	
	Drinks alcohol—less than once a month	0.84 (0.67–1.04)	0.111	0.93 (0.74–1.16)	0.499
	Drinks alcohol—more than once a month	1.02 (0.84–1.23)	0.869	1.08 (0.95–1.23)	0.246
Participates in social activities with friends and relatives	No	1.00 (Reference)		1.00 (Reference)	
	Yes	1.00 (0.88–1.13)	0.959	1.01 (0.89–1.14)	0.884
Participates in recreational and leisure social activities	No	1.00 (Reference)		1.00 (Reference)	
	Yes	1.12 (0.98–1.28)	0.106	1.02 (0.86–1.20)	0.859
Participates in community activities	No	1.00 (Reference)		1.00 (Reference)	
	Yes	1.03 (0.62–1.72)	0.897	0.64 (0.35–1.16)	0.140
Participates in volunteer/charity activities	No	1.00 (Reference)		1.00 (Reference)	
	Yes	1.10 (0.81–1.49)	0.549	1.81 (1.18–2.77)	0.007
Participates in school/training activities	No	1.00 (Reference)		1.00 (Reference)	
	Yes	1.22 (0.73–2.03)	0.443	0.63 (0.23–1.73)	0.365
Internet usage status	Does not use the Internet	1.00 (Reference)		1.00 (Reference)	
	Uses the Internet	0.80 (0.69–0.93)	0.003	1.01 (0.88–1.15)	0.909

Sex-specific models use the full DAG adjustment set except sex. Formal effect-modification tests are reported below.

**Table 9:** Age-specific associations with elevated depressive symptoms and life satisfaction.

**Panel A. Elevated Depressive Symptoms**							
**Variable**	**Level**	**45–59 Years**		**60–74 Years**		**≥75 Years**	
		**PR (95% CI)**	***p* Value**	**PR (95% CI)**	***p* Value**	**PR (95% CI)**	***p* Value**
Total daily sleep duration	7–9 h	1.00 (Reference)		1.00 (Reference)		1.00 (Reference)	
	<7 h	1.63 (1.36–1.95)	<0.001	1.37 (1.23–1.54)	<0.001	1.47 (1.08–2.00)	0.014
	>9 h	1.44 (0.92–2.26)	0.110	0.93 (0.72–1.21)	0.607	1.06 (0.70–1.61)	0.786
Physical activity sufficiency	<600 MET-min/week	1.00 (Reference)		1.00 (Reference)		1.00 (Reference)	
	≥600 MET-min/week	1.25 (1.01–1.56)	0.039	1.03 (0.90–1.18)	0.678	1.68 (1.20–2.36)	0.003
Smoking status	Non-smoker	1.00 (Reference)		1.00 (Reference)		1.00 (Reference)	
	Smoker	1.01 (0.76–1.35)	0.921	1.12 (0.99–1.27)	0.073	0.99 (0.70–1.41)	0.965
Alcohol consumption status	Does not drink alcohol	1.00 (Reference)		1.00 (Reference)		1.00 (Reference)	
	Drinks alcohol—less than once a month	1.02 (0.76–1.37)	0.900	1.14 (0.92–1.41)	0.229	1.03 (0.68–1.57)	0.888
	Drinks alcohol—more than once a month	0.86 (0.73–1.01)	0.059	0.90 (0.78–1.03)	0.117	0.81 (0.57–1.16)	0.253
Participates in social activities with friends and relatives	No	1.00 (Reference)		1.00 (Reference)		1.00 (Reference)	
	Yes	0.95 (0.83–1.09)	0.472	1.08 (0.98–1.18)	0.116	0.94 (0.73–1.20)	0.602
Participates in recreational and leisure social activities	No	1.00 (Reference)		1.00 (Reference)		1.00 (Reference)	
	Yes	0.82 (0.68–0.99)	0.041	0.88 (0.77–1.01)	0.069	0.90 (0.64–1.26)	0.531
Participates in community activities	No	1.00 (Reference)		1.00 (Reference)		1.00 (Reference)	
	Yes	1.23 (0.86–1.77)	0.258	0.65 (0.44–0.96)	0.033	1.09 (0.41–2.92)	0.858
Participates in volunteer/charity activities	No	1.00 (Reference)		1.00 (Reference)		1.00 (Reference)	
	Yes	0.89 (0.69–1.15)	0.364	1.06 (0.80–1.41)	0.696	1.13 (0.50–2.55)	0.772
Participates in school/training activities	No	1.00 (Reference)		1.00 (Reference)		1.00 (Reference)	
	Yes	0.95 (0.63–1.43)	0.791	0.75 (0.38–1.46)	0.394	0.69 (0.14–3.49)	0.654
Internet usage status	Does not use the Internet	1.00 (Reference)		1.00 (Reference)		1.00 (Reference)	
	Uses the Internet	0.98 (0.88–1.09)	0.751	0.96 (0.83–1.10)	0.543	1.41 (0.74–2.67)	0.294
**Panel B. Life Satisfaction**							
**Variable**	**Level**	**45–59 Years**		**60–74 Years**		**≥75 Years**	
		**OR (95% CI)**	***p* Value**	**OR (95% CI)**	***p* Value**	**OR (95% CI)**	***p* Value**
Total daily sleep duration	7–9 h	1.00 (Reference)		1.00 (Reference)		1.00 (Reference)	
	<7 h	0.68 (0.59–0.79)	<0.001	0.75 (0.66–0.85)	<0.001	0.90 (0.68–1.20)	0.472
	>9 h	1.18 (0.94–1.47)	0.148	1.10 (0.88–1.39)	0.401	1.44 (0.94–2.20)	0.095
Physical activity sufficiency	<600 MET-min/week	1.00 (Reference)		1.00 (Reference)		1.00 (Reference)	
	≥600 MET-min/week	1.05 (0.80–1.36)	0.746	1.00 (0.83–1.21)	0.999	0.72 (0.52–1.01)	0.055
Smoking status	Non-smoker	1.00 (Reference)		1.00 (Reference)		1.00 (Reference)	
	Smoker	0.91 (0.71–1.15)	0.420	1.03 (0.88–1.21)	0.714	1.02 (0.74–1.41)	0.912
Alcohol consumption status	Does not drink alcohol	1.00 (Reference)		1.00 (Reference)		1.00 (Reference)	
	Drinks alcohol—less than once a month	0.89 (0.69–1.14)	0.357	0.82 (0.66–1.01)	0.057	1.03 (0.65–1.64)	0.888
	Drinks alcohol—more than once a month	0.97 (0.82–1.14)	0.701	1.12 (0.97–1.30)	0.123	1.22 (0.88–1.70)	0.234
Participates in social activities with friends and relatives	No	1.00 (Reference)		1.00 (Reference)		1.00 (Reference)	
	Yes	0.97 (0.82–1.15)	0.707	1.04 (0.93–1.17)	0.503	1.06 (0.79–1.43)	0.693
Participates in recreational and leisure social activities	No	1.00 (Reference)		1.00 (Reference)		1.00 (Reference)	
	Yes	1.03 (0.88–1.21)	0.717	1.08 (0.93–1.26)	0.317	1.09 (0.79–1.50)	0.594
Participates in community activities	No	1.00 (Reference)		1.00 (Reference)		1.00 (Reference)	
	Yes	0.81 (0.40–1.62)	0.547	0.83 (0.56–1.22)	0.338	0.73 (0.36–1.51)	0.399
Participates in volunteer/charity activities	No	1.00 (Reference)		1.00 (Reference)		1.00 (Reference)	
	Yes	1.78 (1.16–2.74)	0.008	1.06 (0.71–1.57)	0.783	1.27 (0.58–2.79)	0.555
Participates in school/training activities	No	1.00 (Reference)		1.00 (Reference)		1.00 (Reference)	
	Yes	0.65 (0.31–1.40)	0.272	1.26 (0.72–2.21)	0.417	2.93 (0.37–23.43)	0.310
Internet usage status	Does not use the Internet	1.00 (Reference)		1.00 (Reference)		1.00 (Reference)	
	Uses the Internet	0.76 (0.65–0.89)	<0.001	1.09 (0.92–1.28)	0.327	0.95 (0.60–1.49)	0.815

Age-specific models use the full DAG adjustment set except age group. Formal effect-modification tests are reported in Table 10.

**Table 10:** Wald tests for interaction by sex and age group.

Effect Modifier	Outcome	Exposure	df	Wald F	P for Interaction
Sex	Elevated depressive symptoms	Sleep duration	2	0.55	0.577
Sex	Life satisfaction	Sleep duration	2	1.09	0.336
Age group	Elevated depressive symptoms	Sleep duration	4	0.86	0.487
Age group	Life satisfaction	Sleep duration	4	1.49	0.204
Sex	Elevated depressive symptoms	Physical activity	1	1.75	0.187
Sex	Life satisfaction	Physical activity	1	5.08	0.025
Age group	Elevated depressive symptoms	Physical activity	2	3.71	0.025
Age group	Life satisfaction	Physical activity	2	2.14	0.119
Sex	Elevated depressive symptoms	Smoking	1	0.31	0.580
Sex	Life satisfaction	Smoking	1	1.77	0.184
Age group	Elevated depressive symptoms	Smoking	2	0.48	0.621
Age group	Life satisfaction	Smoking	2	0.82	0.440
Sex	Elevated depressive symptoms	Alcohol consumption	2	2.19	0.113
Sex	Life satisfaction	Alcohol consumption	2	0.61	0.544
Age group	Elevated depressive symptoms	Alcohol consumption	4	0.10	0.984
Age group	Life satisfaction	Alcohol consumption	4	0.97	0.422
Sex	Elevated depressive symptoms	Friends/relatives activities	1	0.10	0.753
Sex	Life satisfaction	Friends/relatives activities	1	0.51	0.473
Age group	Elevated depressive symptoms	Friends/relatives activities	2	0.88	0.416
Age group	Life satisfaction	Friends/relatives activities	2	0.43	0.651
Sex	Elevated depressive symptoms	Recreational/leisure activities	1	0.10	0.750
Sex	Life satisfaction	Recreational/leisure activities	1	0.19	0.666
Age group	Elevated depressive symptoms	Recreational/leisure activities	2	0.91	0.405
Age group	Life satisfaction	Recreational/leisure activities	2	0.42	0.655
Sex	Elevated depressive symptoms	Community activities	1	0.41	0.521
Sex	Life satisfaction	Community activities	1	2.30	0.130
Age group	Elevated depressive symptoms	Community activities	2	3.07	0.047
Age group	Life satisfaction	Community activities	2	0.21	0.808
Sex	Elevated depressive symptoms	Volunteer/charity activities	1	0.29	0.590
Sex	Life satisfaction	Volunteer/charity activities	1	2.63	0.106
Age group	Elevated depressive symptoms	Volunteer/charity activities	2	0.55	0.577
Age group	Life satisfaction	Volunteer/charity activities	2	1.33	0.265
Sex	Elevated depressive symptoms	School/training activities	1	0.28	0.594
Sex	Life satisfaction	School/training activities	1	0.89	0.347
Age group	Elevated depressive symptoms	School/training activities	2	0.36	0.697
Age group	Life satisfaction	School/training activities	2	1.31	0.270
Sex	Elevated depressive symptoms	Internet use	1	0.70	0.403
Sex	Life satisfaction	Internet use	1	6.42	0.012
Age group	Elevated depressive symptoms	Internet use	2	0.89	0.411
Age group	Life satisfaction	Internet use	2	5.05	0.007

Each interaction was evaluated in a separate survey-weighted model using the corresponding full DAG adjustment set.

## Data Availability

The data used in this study were obtained from the China Health and Retirement Longitudinal Study (CHARLS), which is managed by Peking University. The CHARLS datasets are publicly available to registered researchers through the official CHARLS website: https://charls.pku.edu.cn/ (accessed on 13 August 2026). The authors do not own the data and are not authorized to redistribute them.

## Supplementary Materials

The following supporting information can be downloaded at: https://www.mdpi.com/article/10.3390/bs16081455/s1: Table S1: Sensitivity analyses comparing baseline-adjusted and fully adjusted DAG models; Table S2: Digital participation behaviors and associations with depressive symptoms and life satisfaction; Table S3: Sensitivity analysis of sleep duration; Table S4: Comparison of included and excluded participants.
