# Supplementary material for: A Study on the Depressive Symptoms and Life Satisfaction Among Middle-Aged and Older Adults in China Based on CHARLS 2020 Data
Source: Behav Sci (Basel). 2026 Aug 21;16(8):1455. doi: 10.3390/bs16081455 (PMC13510261; doi:10.3390/bs16081455)
Supplement: Supplementary file 1 [file behavsci-16-01455-s001.zip › behavsci-4416930-supplementary.pdf]

**Supplementary Table S1: Sensitivity analyses comparing baseline-adjusted and fully adjusted DAG models**

**Panel A. Elevated depressive symptoms**

| Variable | Level | Basic adjustment | | Full adjustment | |
|---|---|---|---|---|---|
| | | PR (95% CI) | P value | PR (95% CI) | P value |
| Total daily sleep duration | 7–9 h | 1.00 (Reference) | | 1.00 (Reference) | |
| | <7 h | 1.69 (1.51–1.89) | <0.001 | 1.49 (1.33–1.68) | <0.001 |
| | >9 h | 1.14 (0.94–1.39) | 0.173 | 1.13 (0.93–1.37) | 0.232 |
| Physical activity sufficiency | <600 MET-min/week | 1.00 (Reference) | | 1.00 (Reference) | |
| | >=600 MET-min/week | 0.95 (0.84–1.07) | 0.404 | 1.17 (1.04–1.32) | 0.009 |
| Smoking status | Non-smoker | 1.00 (Reference) | | 1.00 (Reference) | |
| | Smoker | 0.99 (0.86–1.15) | 0.916 | 1.05 (0.91–1.22) | 0.490 |
| Alcohol consumption status | Does not drink alcohol | 1.00 (Reference) | | 1.00 (Reference) | |
| | Drinks alcohol - less than once a month | 1.01 (0.86–1.20) | 0.870 | 1.06 (0.90–1.25) | 0.503 |
| | Drinks alcohol - more than once a month | 0.80 (0.72–0.89) | <0.001 | 0.86 (0.77–0.95) | 0.004 |
| Participates in social activities with friends and relatives | No | 1.00 (Reference) | | 1.00 (Reference) | |
| | Yes | 1.00 (0.93–1.08) | 1.000 | 1.01 (0.93–1.08) | 0.897 |
| Participates in recreational and leisure social activities | No | 1.00 (Reference) | | 1.00 (Reference) | |
| | Yes | 0.83 (0.74–0.92) | <0.001 | 0.86 (0.77–0.96) | 0.008 |
| Participates in community activities | No | 1.00 (Reference) | | 1.00 (Reference) | |
| | Yes | 0.93 (0.70–1.25) | 0.643 | 0.92 (0.69–1.22) | 0.556 |
| Participates in volunteer/charity activities | No | 1.00 (Reference) | | 1.00 (Reference) | |
| | Yes | 1.00 (0.83–1.21) | 0.986 | 0.96 (0.80–1.16) | 0.688 |

| | | | | | |
|---|---|---|---|---|---|
| Participates in school/training activities | No | 1.00 (Reference) | | 1.00 (Reference) | |
| | Yes | 0.84 (0.61–1.17) | 0.299 | 0.88 (0.64–1.23) | 0.462 |
| Internet usage status | Does not use the Internet | 1.00 (Reference) | | 1.00 (Reference) | |
| | Uses the Internet | 0.92 (0.83–1.02) | 0.115 | 0.98 (0.90–1.08) | 0.712 |
| Sex | Female | 1.00 (Reference) | | 1.00 (Reference) | |
| | Male | 0.77 (0.69–0.86) | <0.001 | 0.77 (0.70–0.86) | <0.001 |
| Age group | 45–59 | 1.00 (Reference) | | 1.00 (Reference) | |
| | 60-74 | 1.02 (0.88–1.18) | 0.798 | 0.87 (0.75–1.00) | 0.059 |
| | 75+ | 0.93 (0.72–1.20) | 0.584 | 0.75 (0.58–0.96) | 0.022 |
| Income quintile | Lowest-income group | 1.00 (Reference) | | 1.00 (Reference) | |
| | Q2 (lower-middle income) | 1.00 (0.90–1.12) | 0.961 | 0.98 (0.87–1.09) | 0.664 |
| | Q3 (middle income) | 1.02 (0.91–1.14) | 0.732 | 1.02 (0.91–1.15) | 0.682 |
| | Q4 (upper-middle income) | 0.70 (0.58–0.84) | <0.001 | 0.77 (0.64–0.93) | 0.006 |
| | Q5 (highest income) | 0.54 (0.41–0.72) | <0.001 | 0.60 (0.46–0.79) | <0.001 |
| Region | Northeast region | 1.00 (Reference) | | 1.00 (Reference) | |
| | Eastern region | 0.97 (0.78–1.22) | 0.797 | 1.07 (0.87–1.33) | 0.507 |
| | Central region | 1.31 (1.10–1.56) | 0.003 | 1.34 (1.15–1.56) | <0.001 |
| | Western region | 1.24 (1.04–1.49) | 0.018 | 1.24 (1.06–1.46) | 0.008 |
| Marital status | Not married | 1.00 (Reference) | | 1.00 (Reference) | |
| | Married | 0.85 (0.77–0.94) | 0.001 | 0.91 (0.83–0.99) | 0.030 |
| Medical insurance | Not participating in medical insurance | 1.00 (Reference) | | 1.00 (Reference) | |
| | Residential medical insurance | 0.86 (0.73–1.01) | 0.062 | 0.92 (0.78–1.08) | 0.296 |

| | | | | | |
|---|---|---|---|---|---|
| | Enterprise employee medical insurance | 0.85 (0.65–1.13) | 0.266 | 0.87 (0.66–1.14) | 0.313 |
| | Public medical care | 0.65 (0.35–1.21) | 0.175 | 0.67 (0.38–1.21) | 0.187 |
| Education level | Primary school and lower | 1.00 (Reference) | | 1.00 (Reference) | |
| | Education-Secondary school | 0.85 (0.73–0.99) | 0.038 | 0.91 (0.77–1.07) | 0.269 |
| | Education-Junior college and above | 0.57 (0.35–0.92) | 0.022 | 0.63 (0.40–0.99) | 0.044 |
| Residence | Rural | 1.00 (Reference) | | 1.00 (Reference) | |
| | Urban | 0.85 (0.75–0.97) | 0.015 | 0.89 (0.78–1.01) | 0.065 |
| Household members | Per 1-unit increase | 0.98 (0.95–1.00) | 0.086 | 0.98 (0.96–1.01) | 0.139 |
| Number of living children | Per 1-unit increase | 1.02 (0.98–1.06) | 0.364 | 1.01 (0.97–1.04) | 0.743 |
| Number of chronic diseases | Per 1-unit increase | | | 1.10 (1.08–1.13) | <0.001 |
| ADL limitations | Per 1-unit increase | | | 1.09 (1.06–1.13) | <0.001 |
| IADL limitations | Per 1-unit increase | | | 1.14 (1.11–1.18) | <0.001 |
| Core cognition score | Per 1-unit increase | | | 0.97 (0.96–0.98) | <0.001 |

**Panel B. Life satisfaction**

| Variable | Level | Basic adjustment | | Full adjustment | |
|---|---|---|---|---|---|
| | | OR (95% CI) | P value | OR (95% CI) | P value |
| Total daily sleep duration | 7–9 h | 1.00 (Reference) | | 1.00 (Reference) | |
| | <7 h | 0.67 (0.61–0.73) | <0.001 | 0.73 (0.67–0.79) | <0.001 |
| | >9 h | 1.14 (0.98–1.32) | 0.085 | 1.16 (1.00–1.34) | 0.051 |
| Physical activity sufficiency | <600 MET-min/week | 1.00 (Reference) | | 1.00 (Reference) | |
| | >=600 MET-min/week | 1.12 (0.94–1.32) | 0.213 | 0.96 (0.81–1.15) | 0.667 |
| Smoking status | Non-smoker | 1.00 (Reference) | | 1.00 (Reference) | |
| | Smoker | 1.01 (0.90–1.14) | 0.828 | 0.97 (0.85–1.10) | 0.605 |

| | | | | | |
|---|---|---|---|---|---|
| **Alcohol consumption status** | **Does not drink alcohol** | **1.00 (Reference)** | | **1.00 (Reference)** | |
| | **Drinks alcohol - less than once a month** | **0.90 (0.77–1.05)** | **0.168** | **0.88 (0.75–1.03)** | **0.105** |
| | **Drinks alcohol - more than once a month** | **1.10 (0.99–1.23)** | **0.068** | **1.05 (0.94–1.17)** | **0.368** |
| **Participates in social activities with friends and relatives** | **No** | **1.00 (Reference)** | | **1.00 (Reference)** | |
| | **Yes** | **1.01 (0.92–1.11)** | **0.896** | **1.01 (0.92–1.11)** | **0.886** |
| **Participates in recreational and leisure social activities** | **No** | **1.00 (Reference)** | | **1.00 (Reference)** | |
| | **Yes** | **1.08 (0.98–1.20)** | **0.125** | **1.06 (0.95–1.18)** | **0.290** |
| **Participates in community activities** | **No** | **1.00 (Reference)** | | **1.00 (Reference)** | |
| | **Yes** | **0.81 (0.49–1.34)** | **0.406** | **0.80 (0.47–1.37)** | **0.418** |
| **Participates in volunteer/charity activities** | **No** | **1.00 (Reference)** | | **1.00 (Reference)** | |
| | **Yes** | **1.39 (1.06–1.82)** | **0.017** | **1.43 (1.09–1.87)** | **0.010** |
| **Participates in school/training activities** | **No** | **1.00 (Reference)** | | **1.00 (Reference)** | |
| | **Yes** | **0.82 (0.42–1.59)** | **0.550** | **0.81 (0.41–1.61)** | **0.552** |
| **Internet usage status** | **Does not use the Internet** | **1.00 (Reference)** | | **1.00 (Reference)** | |
| | **Uses the Internet** | **0.90 (0.81–0.99)** | **0.035** | **0.90 (0.81–1.00)** | **0.045** |
| **Sex** | **Female** | **1.00 (Reference)** | | **1.00 (Reference)** | |
| | **Male** | **0.90 (0.80–1.01)** | **0.082** | **0.89 (0.79–1.00)** | **0.058** |
| **Age group** | **45–59** | **1.00 (Reference)** | | **1.00 (Reference)** | |
| | **60-74** | **1.01 (0.91–1.12)** | **0.857** | **1.13 (1.01–1.26)** | **0.029** |
| | **75+** | **1.20 (0.98–1.46)** | **0.074** | **1.42 (1.17–1.74)** | **<0.001** |
| **Income quintile** | **Lowest-income group** | **1.00 (Reference)** | | **1.00 (Reference)** | |

| | | | | | |
|---|---|---|---|---|---|
| | **Q2 (lower-middle income)** | **1.10 (0.95–1.28)** | **0.190** | **1.14 (0.98–1.32)** | **0.084** |
| | **Q3 (middle income)** | **1.15 (1.01–1.32)** | **0.040** | **1.18 (1.03–1.34)** | **0.016** |
| | **Q4 (upper-middle income)** | **1.22 (1.06–1.39)** | **0.005** | **1.16 (1.01–1.33)** | **0.036** |
| | **Q5 (highest income)** | **1.41 (1.20–1.65)** | **<0.001** | **1.34 (1.14–1.58)** | **<0.001** |
| **Region** | **Northeast region** | **1.00 (Reference)** | | **1.00 (Reference)** | |
| | **Eastern region** | **0.83 (0.67–1.03)** | **0.084** | **0.78 (0.63–0.96)** | **0.019** |
| | **Central region** | **0.80 (0.65–0.97)** | **0.026** | **0.79 (0.65–0.96)** | **0.015** |
| | **Western region** | **0.82 (0.66–1.01)** | **0.067** | **0.82 (0.67–1.00)** | **0.051** |
| **Marital status** | **Not married** | **1.00 (Reference)** | | **1.00 (Reference)** | |
| | **Married** | **1.11 (0.97–1.27)** | **0.116** | **1.09 (0.95–1.25)** | **0.199** |
| **Medical insurance** | **Not participating in medical insurance** | **1.00 (Reference)** | | **1.00 (Reference)** | |
| | **Residential medical insurance** | **1.40 (1.10–1.78)** | **0.006** | **1.33 (1.05–1.68)** | **0.018** |
| | **Enterprise employee medical insurance** | **1.44 (1.09–1.90)** | **0.010** | **1.43 (1.09–1.88)** | **0.009** |
| | **Public medical care** | **2.45 (1.35–4.44)** | **0.003** | **2.37 (1.29–4.37)** | **0.006** |
| **Education level** | **Primary school and lower** | **1.00 (Reference)** | | **1.00 (Reference)** | |
| | **Education-Secondary school** | **0.79 (0.71–0.88)** | **<0.001** | **0.79 (0.70–0.88)** | **<0.001** |
| | **Education-Junior college and above** | **0.90 (0.63–1.28)** | **0.553** | **0.88 (0.61–1.27)** | **0.494** |
| **Residence** | **Rural** | **1.00 (Reference)** | | **1.00 (Reference)** | |
| | **Urban** | **0.92 (0.81–1.05)** | **0.219** | **0.91 (0.80–1.04)** | **0.165** |
| **Household members** | **Per 1-unit increase** | **1.07 (1.03–1.10)** | **<0.001** | **1.07 (1.03–1.10)** | **<0.001** |

| | | | | | |
|---|---|---|---|---|---|
| **Number of living children** | **Per 1-unit increase** | **1.06 (1.02–1.11)** | **0.003** | **1.07 (1.03–1.11)** | **0.002** |
| **Number of chronic diseases** | **Per 1-unit increase** | | | **0.92 (0.90–0.94)** | **<0.001** |
| **ADL limitations** | **Per 1-unit increase** | | | **0.83 (0.78–0.88)** | **<0.001** |
| **IADL limitations** | **Per 1-unit increase** | | | **0.86 (0.82–0.91)** | **<0.001** |
| **Core cognition score** | **Per 1-unit increase** | | | **0.99 (0.98–1.01)** | **0.319** |

***Basic adjustment includes demographic and socioeconomic/contextual covariates. Full adjustment additionally includes chronic disease count, ADL, IADL and cognition.***

**Supplementary Table S2: Digital participation behaviors and associations with depressive symptoms and life satisfaction**

| Variable | Level | Elevated depressive symptoms | | Life satisfaction | |
|---|---|---|---|---|---|
| | | PR (95% CI) | P value | OR (95% CI) | P value |
| Internet use - Chatting | Does not chat online | 1.00 (Reference) | | 1.00 (Reference) | |
| | Chats online | 0.81 (0.64–1.02) | 0.074 | 1.14 (0.96–1.35) | 0.123 |
| Internet use - Reading news | Does not browse news online | 1.00 (Reference) | | 1.00 (Reference) | |
| | Browses news online | 0.90 (0.78–1.03) | 0.134 | 0.94 (0.78–1.15) | 0.564 |
| Internet use - Watching videos | Does not watch videos online | 1.00 (Reference) | | 1.00 (Reference) | |
| | Watches videos online | 0.87 (0.76–0.99) | 0.042 | 1.08 (0.90–1.30) | 0.386 |
| Internet use - Plays games | Does not play games online | 1.00 (Reference) | | 1.00 (Reference) | |
| | Plays games online | 0.65 (0.50–0.83) | <0.001 | 1.29 (1.01–1.64) | 0.043 |
| Mobile payment | Does not use mobile payment | 1.00 (Reference) | | 1.00 (Reference) | |
| | Uses mobile payment | 0.91 (0.78–1.07) | 0.277 | 0.75 (0.65–0.87) | <0.001 |
| WeChat usage | Does not use WeChat | 1.00 (Reference) | | 1.00 (Reference) | |
| | Uses WeChat | 1.11 (0.93–1.33) | 0.245 | 0.80 (0.65–0.99) | 0.043 |
| WeChat Moments posting | Does not post on WeChat Moments | 1.00 (Reference) | | 1.00 (Reference) | |
| | Posts on WeChat Moments | 0.95 (0.82–1.10) | 0.529 | 0.92 (0.78–1.07) | 0.265 |

***Each digital behavior was assessed in a separate survey-weighted model adjusted for demographic, socioeconomic/contextual, chronic disease, ADL, IADL and cognition variables.***

**Supplementary Table S3： Sensitivity analysis of sleep duration; Table S4: Comparison of included and excluded participants**

| Variable | Level | Primary model | | Sensitivity model | |
|---|---|---|---|---|---|
| | | PR (95% CI) | P value | PR (95% CI) | P value |
| Total daily sleep duration | 7–9 h | 1.00 (Reference) | | 1.00 (Reference) | |
| | <7 h | 1.49 (1.33–1.68) | <0.001 | 1.49 (1.32–1.68) | <0.001 |
| | >9 h | 1.13 (0.93–1.37) | 0.232 | 1.12 (0.93–1.36) | 0.239 |

***Both survey-weighted modified Poisson models use the full DAG adjustment set. Analysis n: primary model 13,576; sensitivity model 13,490.***
